# Multiple Single-Cell Genomes Provide Insight into Functions of Uncultured *Deltaproteobacteria* in the Human Oral Cavity

**DOI:** 10.1371/journal.pone.0059361

**Published:** 2013-03-26

**Authors:** Alisha G. Campbell, James H. Campbell, Patrick Schwientek, Tanja Woyke, Alexander Sczyrba, Steve Allman, Clifford J. Beall, Ann Griffen, Eugene Leys, Mircea Podar

**Affiliations:** 1 Genome Science and Technology Program, University of Tennessee, Knoxville, Tennessee, United States of America; 2 Biosciences Division, Oak Ridge National Laboratories, Oak Ridge, Tennessee, United States of America; 3 DOE Joint Genome Institute, Walnut Creek, California, United States of America; 4 College of Dentistry, Ohio State University, Columbus, Ohio, United States of America; 5 Department of Microbiology, University of Tennessee, Knoxville, Tennessee, United States of America; University of Vienna, Austria

## Abstract

Despite a long history of investigation, many bacteria associated with the human oral cavity have yet to be cultured. Studies that correlate the presence or abundance of uncultured species with oral health or disease highlight the importance of these community members. Thus, we sequenced several single-cell genomic amplicons from *Desulfobulbus* and *Desulfovibrio* (class *Deltaproteobacteria*) to better understand their function within the human oral community and their association with periodontitis, as well as other systemic diseases. Genomic data from oral *Desulfobulbus* and *Desulfovibrio* species were compared to other available deltaproteobacterial genomes, including from a subset of host-associated species. While both groups share a large number of genes with other environmental *Deltaproteobacteria* genomes, they encode a wide array of unique genes that appear to function in survival in a host environment. Many of these genes are similar to virulence and host adaptation factors of known human pathogens, suggesting that the oral *Deltaproteobacteria* have the potential to play a role in the etiology of periodontal disease.

## Introduction

Recent large-scale efforts have profiled the large number of microbial communities associated with the human body and the importance of determining the composition and function of these communities and, ultimately, their effects on human health [1]–[3]. Studies of microbes in the human oral cavity have been ongoing since the discovery of “animalcules” by Antony van Leeuwenhoek in 1676, but despite this long history, over 50% of oral microbes remains uncultured. Likewise, periodontitis, an inflammatory gum disease, has been under investigation since the early 19^th^ century, but the role of microbial communities associated with the disease remains unclear [4], [5]. Periodontitis is the leading cause of tooth loss worldwide and has been linked to a number of systemic diseases, including diabetes, cardiovascular disease, osteoporosis and preterm low birth weight [6]–[11]. Thus, understanding the roles of periodontitis-associated microbial community members is of utmost importance. Genomic information provides an initial way to assess functional potential. Although there are relatively few published analyses using closed genome sequences of cultured bacteria associated with periodontitis [12]–[14], under the Human Microbiome Project (HMP) there are over 400 ongoing or completed genome sequencing projects for cultured human oral bacteria [2] (http://www.hmpdacc.org/). Combined with the hundreds of oral metagenomic datasets generated by the HMP [15], this vast genomic information for health- and disease-associated microbes should ultimately result in more effective treatments and preventative measures for periodontitis and other oral diseases.

Recent culture-independent studies have associated several uncultured organisms with periodontitis [16]–[19]. One group of uncultured oral microbes that are of great importance is sulfate-reducing bacteria (SRB). These organisms have been of long-standing interest because of their ability to produce hydrogen sulfide, a compound that can be toxic to human cells. Potential SRB within the oral microbial community include several members of *Deltaproteobacteria*. Both culture-based studies [20], [21] as well as quantitative PCR studies based upon dissimilatory sulfate reduction genes (*dsrAB*) [22] have linked SRB to periodontitis, particularly to more clinically severe cases with deeper periodontal pockets.

Several culture-independent studies have linked the deltaproteobacterial genus *Desulfobulbus*, with progressive periodontitis [17], [23]. *Desulfobulbus* sp. oral taxon 041 is present in low levels in most adults [24] but has been found in greater abundance in both healthy and diseased sites of subjects with periodontitis compared to healthy control subjects [16]. Despite these findings, little is known about oral *Desulfobulbus* species because there are no cultured representatives from this environment and only one genome published from this genus [25]. Isolates of *Desulfovibrio fairfieldensis* and closely related species have also been associated with periodontitis [26], and these organisms have been found as the causative agent of several cases of bacteremia [27], [28]. Although cultured isolates are available for *Desulfovibrio fairfieldensis*, no genomic information has been published. Obtaining genomic information for these oral *Deltaproteobacteria* would greatly enhance our knowledge and shed light on their potential function in the etiology of progressive periodontitis.

Thus, our aim was to selectively isolate single cells of oral *Deltaproteobacteria* and sequence their genomes. We sequenced both individuals and groups of single-cell amplicons most closely related to the uncultured *Desulfobulbus* sp. oral taxon 041 [3], as well as a group of single-cell amplicons most closely related to the uncultured *Desulfovibrio* sp. oral taxon 040 [3]. Groups of single-cell amplicons were sequenced to provide more complete genomic information for each genus. Genomic data were analyzed and compared to sequenced, environmental isolates of related organisms and other host-associated, *Deltaproteobacteria* genomes.

## Results and Discussion

### Cell Isolation, Sequencing and Phylogenetic Analysis

Single cells of both oral *Desulfobulbus* (n = 7) and *Desulfovibrio* (n = 5) were isolated from ethanol-fixed samples using a combination of fluorescence *in situ* hybridization (FISH) and flow cytometric cell sorting. FISH was performed using *Deltaproteobacteria*-specific probes [29], [30]. A culture of *Desulfobulbus propionicus* 1pr3 (DSM 2032) was used to test and optimize hybridization conditions, (Figure S1). The genomic DNA of single cells was amplified using multiple displacement amplification (MDA) followed by taxonomic characterization of the single cell amplified genomes (SAGs) by small subunit rRNA (SSU rRNA or 16S rRNA) gene amplification and sequencing. SAGs confirmed to represent target *Deltaproteobacteria* were sequenced individually (Dsb1, Dsb2, Dsb3) or in groups (Dsb4, Dsb5, Dsv1). The confirmed deltaproteobacterial SAGs originated from samples collected from both healthy individuals and individuals with periodontitis (Table 1).

**Table 1:** Metadata and genome statistics for single and multi-cell oral amplicons.

	Genome Assembly						
	Dsb1	Dsb2	Dsb3	Dsb4	Dsb5	Dsb1–5	Dsv1
Number of cells sequenced	1	1	1	2	2	7	5
Donor health status	Healthy	Healthy	Periodontitis	Periodontitis	Periodontitis	Healthy+ Periodontitis	Periodontitis
Assembly size (bp)	455,123	768,341	798,161	748,829	959,378	1,883,075	2,603,557
DNA scaffolds	64	164	231	70	112	349	259
G+C (%)	56.6	57.6	57.2	59	59.7	58.6	59.9
Estimated genome size (Mbp)	1.39	2.09	2.12	3.75	2.26	2.48	2.63
Estimated genome recovey (%)	32.8	36.8	37.6	20.0	42.4	76.0	99.1
CRISPR	2	0	1	2	0	–	0
Genes total number	527	808	860	781	1007	1936	2890
5S rRNA	1	1	1	1	1	1	1
SSU rRNA	1	1	1	1	1	1	1
23S rRNA	1	1	1	1	0	1	1
tRNA genes	10	9	12	16	15	23	36
Other RNA genes	0	0	1	0	0	0	4
Protein coding genes	514	796	844	762	990	1910	2847

**Figure 1:**
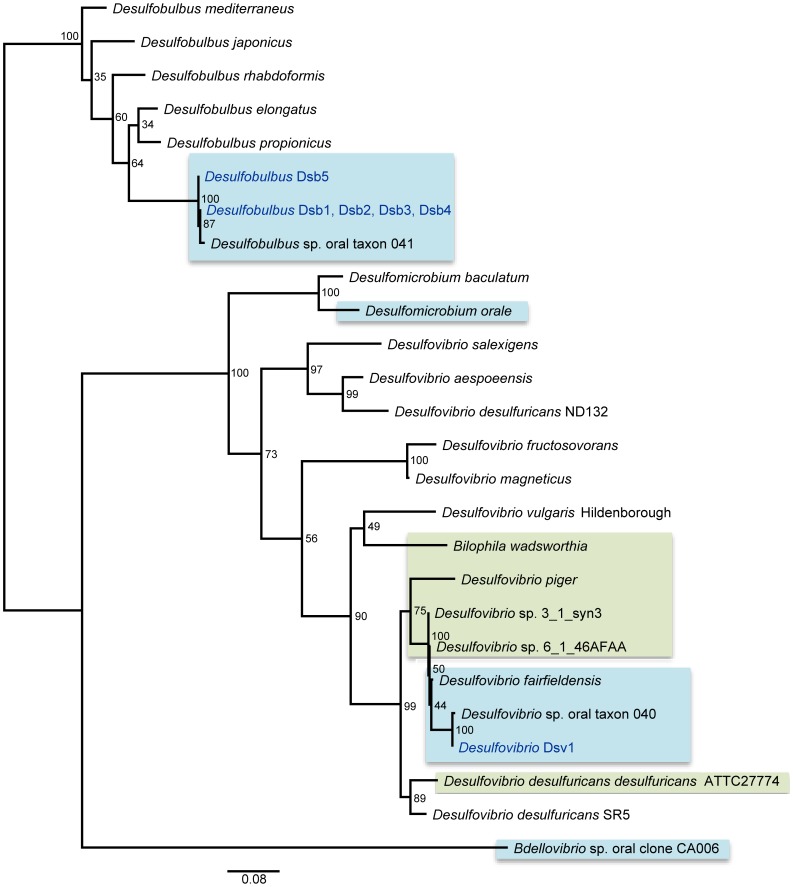
Maximum likelihood phylogenetic tree based on the SSU rRNA gene of select *Deltaproteobacteria* isolates as well as full-length clones from the Human Oral Microbiome Database [**3**]. The tree was reconstructed using PHYML [84] in the program Geneious® Pro 5.6.5 with a GTR (+ gamma+invariant sites) substitution model. Sequences from the present study are in blue text. Other host-associated sequences are denoted by blue (oral) or green (gut/rumen) boxes. The scale bar indicates 0.08 substitutions per nucleotide position. Numbers given at the nodes represent bootstrap percentages calculated on 100 replicates.

Oral *Desulfobulbus* cells sequenced in this study were chosen for genomic sequencing based on SSU rRNA gene similarity to a previously known oral clone, *Desulfobulbus* sp. oral taxon 041 [3], that has been associated with periodontitis [16]. Comparisons of full-length SSU rRNA genes revealed that amplicons Dsb1– Dsb4 are identical, and these amplicons are over 99% identical to Dsb5 and *Desulfobulbus* sp. oral taxon 041 (Figure 1). However, all the oral taxa form a distinct clade compared to *Desulfobulbus* from other environments.

The full length (1430 nt) SSU rRNA gene of Dsv1 is over 99% identical to *Desulfovibrio* sp. oral taxon 040 [3] and 98% identical to *Desulfovibrio fairfieldensis*, a cultured oral isolate [31]. In addition, the SSU rRNA gene of Dsv1 is also highly similar (>98%) to *Desulfovibrio* sp. 3_1_syn3 and *Desulfovibrio* sp. 6_1_46AFAA, two human-associated, sequenced species from the gut (Figure 1). When comparing the predicted proteins of Dsv1 to those of *D.* sp. 3_1_syn3, BLASTP [32] percent identity between the two organisms was 80% or greater for roughly half the proteins predicted in Dsv1. Only 13% of Dsv1 predicted proteins had the same level of similarity to human-associated *Desulfovibrio piger*, and 3% of genes were this closely related to environmental *Desulfovibrio vulgaris* Hildenborough (Figure S2).

### Genome Statistics

After normalization and assembly, the *Desulfobulbus* datasets had 0.45–0.96 Mbp each, with 527-1007 total genes (Table 1). Although Dsb4 and Dsb5 were a combination of two single amplified genomes (SAGs), only Dsb5 showed a noticeable increase in data (Table 1). Higher levels of contamination removal from Dsb4 most likely explain this discrepancy. Analyses comparing average nucleotide identity (ANI) of *Desulfobulbus* data showed that the amplicons were an average of 97.4% similar to one another (Table S1 in File S1). Similarly, comparing tetranucleotide frequencies revealed a 98.7% average similarity between *Desulfobulbus* datasets Dsb1 through Dsb5 (Table S2 in File S1). Although the *Desulfobulbus* cells were isolated from both patients with periodontitis as well as healthy controls, there was no obvious separation between these groups. This similarity is expected since previous studies have shown the same *Desulfobulbus* species present in most adults but increasing in abundance during periodontitis [16], [24] In light of these findings, we treated these cells as members of a single operational taxonomic unit (OTU), and all downstream metabolic analyses were performed on a combined assembly of *Desulfobulbus* data. This combined data set will be referred to as Dsb1-5 throughout. After removing redundant data, Dsb1-5 had a total of 1910 unique genes and 1.9 Mbp, with a GC content of 58.6%. Using a genome size estimation based on a conserved single copy gene set, the genome of this oral *Desulfobulbus* OTU is approximately 2.48 Mbp. Thus, we have captured approximately 76% of the complete genome.

For the oral *Desulfovibrio*, five separate and taxonomically confirmed SAGs were combined before sequencing and will be referred to as Dsv1. The combination of genomic DNA from five SAGs provided a large increase of sequence data compared to the single and two-cell assemblies sequenced for the *Desulfobulbus* species. Dsv1 contained 2.6 Mbp of non-redundant data with a total of 2890 predicted genes and a GC content of 59.9%. A genome size estimate of 2.63 Mbp revealed that we were able to capture over 99% of the *Desulfovibrio* genome. Analysis with BLASTCLUST [32] revealed that the genomic dataset of these five cells, all sorted from the same sample, assembled well. Of the 2842 gene clusters formed at a similarity of 95% and an overlapping length of 0.7, 2839 were unique. The three overlapping clusters contained small, hypothetical genes, with at least one cluster having high similarity to integrase/transposase-like proteins.

### Metabolism

Although Dsb1-5 is human-associated, several aspects of its metabolism appear to be similar to those found in the most closely related sequenced species, *Desulfobulbus. propionicus*, an isolate from anaerobic mud [33]. Forty four percent of Dsb1-5 predicted proteins had top BLASTP [32] hits to proteins in *D. propionicus*. *D. propionicus* has a complete pathway for glycolysis/gluconeogenesis, and most of these genes are also present in Dsb1-5. Similar genes are also found in the pentose phosphate pathway, although several genes were missing in our partial Dsb1-5 genome. The ability to respire sulfate appears to be present in Dsb1-5. The genes for dissimilatory sulfite reductase (*dsrAB*) are present, as well as dissimilatory adenylylsulfate reductase (*aprAB*). In addition, genes encoding QmoA and B and DsrJ, K, M, O and P [34] are present. A dissimilatory nitrate reductase gene is also present in *D. propionicus* and Dsb1-5, although Dsb1-5 had no evidence of the nitrogenase found in *D. propionicus*.

Despite the similarities, key differences can be seen in Dsb1-5 compared to some of the characteristics most associated with the *Desulfobulbus* genus. Several genes needed for propionate metabolism and the methylmalonyl pathway are missing in Dsb1-5 (Figure S3). In particular, genes capable of converting propionyl phosphate to propionyl-CoA are present only in the *D. propionicus* genome. However, Dsb1-5 does code for formate C-acetyltransferase (E.C. 2.3.1.54), an enzyme not seen in *D. propionicus*, which would directly convert 2-oxobutyrate to propionyl-CoA.

Fermentation of pyruvate and alcohols has been found to proceed via the methylmalony-CoA pathway in *D. propionicus* [35]. Two of the three essential genes for the methylmalonyl-CoA fermentation pathway (EC 5.1.99.1 and EC 5.4.99.2) were not found in Dsb1-5, and pyruvate dehydrogenase and malate dehydrogenase, proteins that are active in *D. propionicus* [35], were also absent. Key enzymes of an alternative, acetyl-CoA pathway (formyltetrahydrofolate synthetase, carbon monoxide dehydrogenase and acetyl-CoA synthase) were also absent from Dsb1-5. Although this could be due to the incomplete nature of the Dsb1-5, it allows speculation that these capabilities may have been lost in the human-associated species. Additionally, these genes are not localized in *D. propionicus* but are found across the genome, making it even more unlikely that all such genes would be missed by our sequencing efforts. Dsb1-5 also lacks genes involved in butanoate metabolism and lactate utilization. In addition, *D. propionicus* encodes a partial TCA cycle that includes genes for 2-oxoglutarate:ferrodoxin reductase, but Dsb1-5 has only three genes involved in the TCA cycle, leaving its use of this cycle unclear.

Metabolic capabilities of Dsv1 seem to be similar to those found in other human-associated *Desulfovibrio* genomes (especially *Desulfovibrio* sp. 3_1_syn3), and these genomes also share many similarities to sequenced *Desulfovibrio* species from other environmental niches. Using BLASTP [32], 42% of Dsv1 predicted proteins had top hits to the *Desulfovibrio* sp. 3_1_syn3 genome. Approximately 32% of Dsv1 predicted proteins showed greatest similarity to other host-associated Deltaproteobacteria (*Desulfovibrio* sp. 6_1_46AFAA = 22%, *Desulfovibrio piger* = 2%, *Desulfovibrio desulfuricans* subsp. *desulfuricans* str. ATCC 27774 = 2%, *Bilophila wadsworthia* 3_1_6 = 4%, *Bilophila* sp. 4_1_30 = 2%).

All of the human-associated *Desulfovibrio* species, including Dsv1, contain genes for glycolysis/gluconeogenesis and key components of the pentose phosphate pathway, although the gene for glucose-6-phosphate isomerase is missing in Dsv1. Dsv1 also has the genes necessary for sulfate respiration, including *dsrAB*, *aprAB*, *dsrJKMOP* [34], *qmoABC* and *hmc* genes. All three human-associated *Desulfovibrio* genomes harbor the genes needed to reduce nitrate to ammonia that are also found in many environmental *Desulfovibrio* species. Human-associated *Desulfovibrio* all have an F-type ATPase, cytochrome *bd* complex and cytochrome *c* family III, much like environmental *Desulfovibrio* species. However, there is no evidence for a cytochrome *c* oxidase in the human-associated *Desulfovibrio* species. All host-associated *Desulfovibrio* species have a complete (*D. desulfuricans* ATCC 27774, *D. piger*, Dsv1) or partial (*D.* sp. 3_1_syn3) transporter for methionine that is not seen in any sequenced, environmental *Desulfovibrio* species.

Dsv1 contains an RNF-type complex I similar to other *Desulfovibrio* species. In addition, Dsv1 appears to have a partial Nuo-type NADH dehydrogenase (subunits A-D, H-K) that is not present in either of the gut *Desulfovibrio* genomes, and all subunits have top matches to the NADH dehydrogenase genes from *D. propionicus*. The complex appears to lack the NADH binding and oxidizing subunits (NuoEFG) and may transfer electrons from ferredoxin to quinones of the respiratory chain. Similar complexes were found in Dsb1-5, *D. propionicus* and *D. desulfuricans desulfuricans* ATCC 27774, a bacterium isolated from a sheep rumen. Both Dsv1 and *D.* sp. 3_1_syn3 have genes that encode fumarate reductase, an enzyme that functions in anaerobic respiration, but this appears to be absent in *D. piger*.

#### Chromosome partitioning and cell division

As expected, a gene encoding cell division protein FtsZ was found in Dsb1-5, along with other proteins involved in the cell division protein complex, including FtsA, FtsB, FtsE, FtsK, FtsQ and FtsX. Similar genes encoding the FtsZ system were also found in the Dsv1. In addition, Dsv1 also contained genes encoding the proteins MreB, MreC and RodA, proteins known to be involved in shape determination, particularly for elongated, rod-shaped bacteria.

#### Phage-related genes

Both Dsb1-5 and Dsv1 have large numbers of phage-related genes including Mu-like proteins. Both Dsv1 and Dsb1-5 harbor killer and antidote proteins of a killer gene system. This system can be used for stress response, cell cycle arrest and maintenance of otherwise disposable genes, such as in an integron [36]. All of the toxin/antitoxin gene pairs found in Dsb1-5 and Dsv1 appear to be chromosomal and are found in close proximity to transposase, integrase and other phage-related genes.

#### Replication and Repair

Genes functioning in replication and repair were found in Dsb1-5 and Dsv1, including genes involved in DNA replication, homologous recombination and base excision, mismatch and nucleotide excision repair. The top BLASTP hits for Dsb1-5 were variable, with a wide range of *Deltaproteobacteria* and lower overall percent identity. Most proteins in Dsv1 had high BLASTP percent identity to proteins predicted in two HMP *Desulfovibrio* sp. that have been sequenced from the human gut (*D. sp. 3_1_syn3* and *D. sp. 6_1_46AFAA*). Thus, replication and repair machinery in the oral Dsb1-5 are further removed from any of the currently sequenced *Deltaproteobacteria* than that of Dsv1.

#### Motility

Cell mobility is an important factor of chemotaxis, biofilm formation and oral pathogenicity, and the oral *Deltaproteobacteria* characterized here have the potential for several types of mobility. Dsb1-5 contained proteins for chemotaxis, including methyl-accepting chemotaxis proteins for detection of chemical gradients and chemotactic proteins CheA, CheW and CheY. Dsv1 contained all the chemotaxis proteins found in Dsb1-5, as well as CheB, CheV and CheZ. These chemotactic proteins are involved in a signal transduction system that could control flagellar swimming motility and/or pilin twitching motility.

Evidence for flagellar biosynthetic and motor proteins was present in Dsb1-5 and Dsv1 and included FliG, FliL, FliM, FliN, FliP, FliQ, and motor proteins MotA and MotB. However, only Dsv1 had evidence of flagellar proteins necessary for the formation of the flagellar distal and proximal rods, hook and filament. Although *D. piger* is nonmotile, both Dsv1 and *D.* sp. 3_1_syn3 have all the genes necessary for flagellar motility. *D. propionicus* strains 2pr4 and 3pr10 produce a single polar flagella and are motile while strain 1pr3 was nonmotile without a visible flagellum [33], despite the presence of several flagellar encoding genes. Based on the absence of several flagellar proteins, it is possible that Dsb1-5 has lost the ability to produce flagella, much like *D. propionicus* 1pr3.

In addition to an incomplete set of flagellar proteins, Dsb1-5 also includes a variety of genes for pilin proteins. These genes code for proteins PilA, PilB, PilF, PilO, PilT, PilV, PilW, PilX, and PilY1. The presence of PilT, a protein involved in pilus retraction and disassembly, suggests that Dsb1-5 forms type IV pili that can be used for twitching motility and biofilm formation [37], [38]. This is not surprising as twitching motility is found in organisms within Proteobacteria divisions Delta, Beta and Gamma [37]. *D. propionicus* strain 1pr3 also produces pili [33], and many of the pili genes in Dsb1-5 have top BLASTP hits to *D. propionicus*.

#### Secretion

Several of the genes found in Dsb1-5 for twitching motility also overlap with genes used in type II secretion systems. The presence of additional genes PulD, PulE, PulF and PulO suggest that Dsb1-5 also contain a type II secretion system. Genes that code for both the Sec-dependent protein export as well as twin arginine targeting protein export are present in both Dsb1-5 and Dsv1. A type IV secretion system is also present in both organisms. The type IV proteins match pfam categories that include both virulence and conjugal transfer proteins. The presence of a type IV secretion system potentially allows Dsb1-5 to adapt to environmental changes during periodontitis and to uptake antibiotic resistance genes. It is also possible that conjugative pili could be working to aid colonization and biofilm formation [39]. Both Dsb1-5 and Dsv1 contained a gene encoding a putative homolog of TadD, a Flp pilus assembly protein important for tight, nonspecific adherence of certain bacteria to surfaces [40]. The *tad* locus has been studied in *Aggregatibacter actinomycetemcomitans* and found to be essential for the ability to colonize tooth surfaces [40]. In addition, *tadD* was found to be an important virulence factor in the pathogens *Pasteurella multocida* and *Yersinia ruckeri* [41], [42]. The presence of a membrane fusion protein similar to HlyD points to type I secretion capabilities in both Dsb1-5 and Dsv1.

#### Defense Mechanisms

Several genes in Dsb1-5 are involved in self-defense. It appears that oral *Desulfobulbus* species have both type I and II restriction modification systems in place to protect against bacteriophage. Dsv1 shows evidence of type I and III restriction modification systems. CRISPR regions were found in Dsb1-5, and CRISPR-associated genes were found in both Dsb1-5 and Dsv1. Dsb1-5 and Dsv1 also contain genes that provide a wide range of antibiotic and multi-drug resistance, including both primary and secondary active transporters. Both Multi-Antimicrobial Extrusion (MATE) and Resistance nodulation cell division (RND) families of secondary active transporters were found in Dsb1-5 and Dsv1. One multi-drug efflux pump found in both oral genera had high similarity (0.0e+00 E-value) with the AcrB/D/F family (pfam00873), which is known to have a wide range of substrate specificity and exports most antibiotics in use [43]. In addition, several multidrug and antimicrobial ATP-binding cassette (ABC) transporters are encoded in both Dsb1-5 and Dsv1. Dsb1-5 contains genes for bacteriocin/lantibiotic transporters. Also, oral Dsb1-5 carries class C beta-lactamase genes; thus, it is likely resistant to penicillin and other beta-lactam-containing antibiotics. Finally, Dsb1-5 included an undecaprenyl-diphosphatase gene, which confers resistance to bacitracin.

#### Oxygen-Tolerance

Although originally thought to be obligate anaerobes, it is now known that many sulfate-reducing bacteria are oxygen tolerant. In fact, both *Desulfobulbus* and *Desulfovibrio* species have been shown to perform aerobic respiration with several electron donors, including sulfur compounds. However, these bacteria were not able to grow with oxygen as the electron acceptor [44], but rather, they seem to use aerobic respiration as a defense mechanism to avoid production of toxic products such as hydrogen peroxide and superoxide radicals [45]. Genes important for oxygen tolerance were found in both Dsb1-5 and Dsv1, suggesting that this trait is also important in the oral environment. A catalase gene was found in Dsb1-5, and this enzyme is able to detoxify certain oxygen species [45]. In addition, rubredoxin and desulfoferrodoxin genes were found in both Dsb1-5 and Dsv1, and a gene encoding rubrerythrin was found in Dsb1-5. Homologous genes in *Desulfarculus baarsii* were found to protect against damage caused by reactive oxygen species [46]. The ability to deal with oxygen stress efficiently would be an important trait for successful pathogens.

### Pathogen-associated Genes

Both Dsb1-5 and Dsv1 carry genes that are beneficial for a pathogen lifestyle. Some of these genes are in common with *Desulfovibrio* species found in the environment, but several genes seem to be uniquely associated with a host-associated niche. Pathogen-associated genes provide a microbe with a competitive advantage in a host environment. Putative virulence factors for oral pathogens include genes for adherence, defense, uptake of limited nutrients, stress adaptation and interactions with both host cells and other microbial cells found within a biofilm. Categories of putative virulence factors that were found in Dsb1-5 and Dsv1 are listed in Table 2 and discussed below.

**Table 2:** Categories of putative virulence factors found in Dsb1-5 and Dsv1.

Category	Annotation	Dsb1-5	Dsv1
Acquisition of iron	FeoB ferrous iron uptake	+	+
	ABC-type Fe3+-siderophore transport system	+	+
	Hemolysin	+	+
	ferrous iron transport protein A	+	+
	Fur family ferric uptake regulator	+	+
	TonB-dependent receptor	+	+
Secretion	Sec system	+	+
	Type I secretion	+	+
	Type II secretion	+	
	Type IV secretion	+	+
Stress Response	Universal stress protein UspA	+	+
	Clp proteins	+	+
	RelA/SpoT family protein	+	+
	5'/3'-nucleotidase SurE	+	+
	Crp/Fnr family transcriptional regulator	+	+
	desulfoferrodoxin	+	+
	rubredoxin	+	+
	Rubrerythrin	+	
	Catalase	+	
	Peroxiredoxin		+
Evasion	2-dehydro-3-deoxyphosphooctonate aldolase	+	
	Acetyltransferase	+	+
	alginate o-acetyltransferase	+	
	Beta-glucosidase-related glycosidases		+
	CMP-2-keto-3-deoxyoctulosonic acid synthetase		+
	dTDP-4-dehydrorhamnose 3,5-epimerase		+
	dTDP-4-dehydrorhamnose reductase	+	
	dTDP-glucose 4,6-dehydratase		+
	glucose 1-phosphate thymidylyltransferase	+	
	glycoside hydrolase family protein		
	Glycosyltransferase	+	+
	lytic transglycosylase	+	
	membrane-bound lytic murein transglycosylase		+
	N-acetylmuramoyl-L-alanine amidase	+	+
	NAD-dependent epimerase/dehydratase	+	
	nucleotide sugar dehydrogenase	+	+
	pantetheine-phosphate adenylyltransferase, bacterial		+
	peptidoglycan O-acetyltransferase PacA	+	
	GDP:alpha-D-mannose-1-phosphate guanylyltransferase	+	
	putative exopolysaccharide biosynthesis protein		+
	UDP-N-acetylglucosamine 2-epimerase	+	
	undecaprenyl-phosphate galactose phosphotransferase	+	
Defense mechanism	ABC-type bacteriocin/lantibiotic exporter	+	
	ABC-type multidrug transport system	+	+
	acriflavin resistance protein	+	+
	choline/carnitine/betaine transporter family	+	
	Cation/multidrug efflux pump	+	+
Protease/Peptidase	Clp protease	+	+
	La protease	+	+
	carboxyl-terminal protease	+	
	Collagenase and related proteases		+
	FtsH protease	+	
	Membrane proteins related to metalloendopeptidases		+
	O-sialoglycoprotein endopeptidase	+	
	signal peptide peptidase SppA	+	+
	Trypsin-like serine proteases, typically periplasmic		+
	Xaa-Pro aminopeptidase		+
	putative glycoprotease GCP		+
	periplasmic serine protease, Do/DeqQ family		+
	outer membrane protease	+	
Adhesion	Type IV pili	+	
	TPR repeat-containing protein	+	+
	YD repeat-containing protein	+	
	Surface antigens	+	+
	Lipoproteins	+	+

The presence of a gene is noted by (+).

Iron is an essential element used in several metabolic processes; however, bio-available forms of iron are often limited in the environment [47]. The ability to acquire iron is a particularly important trait for human-associated bacteria, where iron can be even more limited by iron-binding proteins of host cells. In the oral environment, most iron is bound to the host protein lactoferrin [47]. Thus, successful human pathogens often have several genes that give them an advantage in acquiring iron. In addition to several iron transport systems, both Dsb1-5 and Dsv1 also contain hemolysin. Using IMG, these genes can also found in many environmental *Deltaproteobacteria* genomes and have likely persisted in Dsb1-5 and Dsv1 because of their continued benefits in the host environment.

In order to be successful pathogens, bacteria must cope with environmental changes and harmful molecules. Both Dsb1-5 and Dsv1 encode a wide range of transporters and efflux systems to protect against drugs and antibiotics. In addition, Dsb1-5 and Dsv1 contain oxygen-tolerance genes discussed above, universal stress protein UspA and a suite of Clp proteins. The Clp proteins in *Porphyromonas gingivalis* have been shown to be important for stress response, as well as biofilm formation and entry into host epithelial cells [48]. Knockouts of ClpB resulted in reduced virulence in both *Leptospira interrogans* and *Enterococcus faecalis* and reduced general stress resistance in *L. interrogans* [49], [50]. Further, ClpAP mutants in *Helicobacter pylori* showed increased sensitivity to antibiotics and oxidative stress and disrupted colonization of macrophages [51].

Another important aspect of pathogenicity includes the ability to attach to other cells. The type IV pili found in Dsb1-5 are likely important for its adhesion [38], [52]. Additional surface proteins that may play a role in binding to cells include tetratrico peptide repeat (TPR) and YD dipeptide repeat-containing domains, surface antigens and lipoproteins [14]. Additionally, both Dsb1-5 and Dsv1 contain several proteases and peptidases that often serve as virulence factors that degrade host proteins for nutrients and may contribute to the cytotoxicity of periodontitis [14], [53].

Lastly, both genomes had several genes for polysaccharide metabolism, including an abundance of glycosyl transferases [14], [53]. Both genomes had a large number of genes involved in lipopolysaccharide biosynthesis, and there were also genes present in Dsb1-5 (UDP-N-acetylglucosamine 2-epimerase and polysaccharide biosynthesis protein CapD) that are putatively involved in extracellular polysaccharide (EPS) or capsular production. At least two scaffolds in Dsb1-5 include genes that may be involved in EPS or capsular production, and the majority of genes on these scaffolds appear to be horizontally transferred, with most genes having top BLASTP hits to genomes other than *D. propionicus* (Table S3 in File S1). The presence of a capsule would be beneficial to a pathogen in the oral environment by providing adherence to oral surfaces and other biofilm members and resistance to both specific and nonspecific host immune systems [54].

### COG Comparisons to Other *Deltaproteobacteria* Genomes

**Figure 2:**
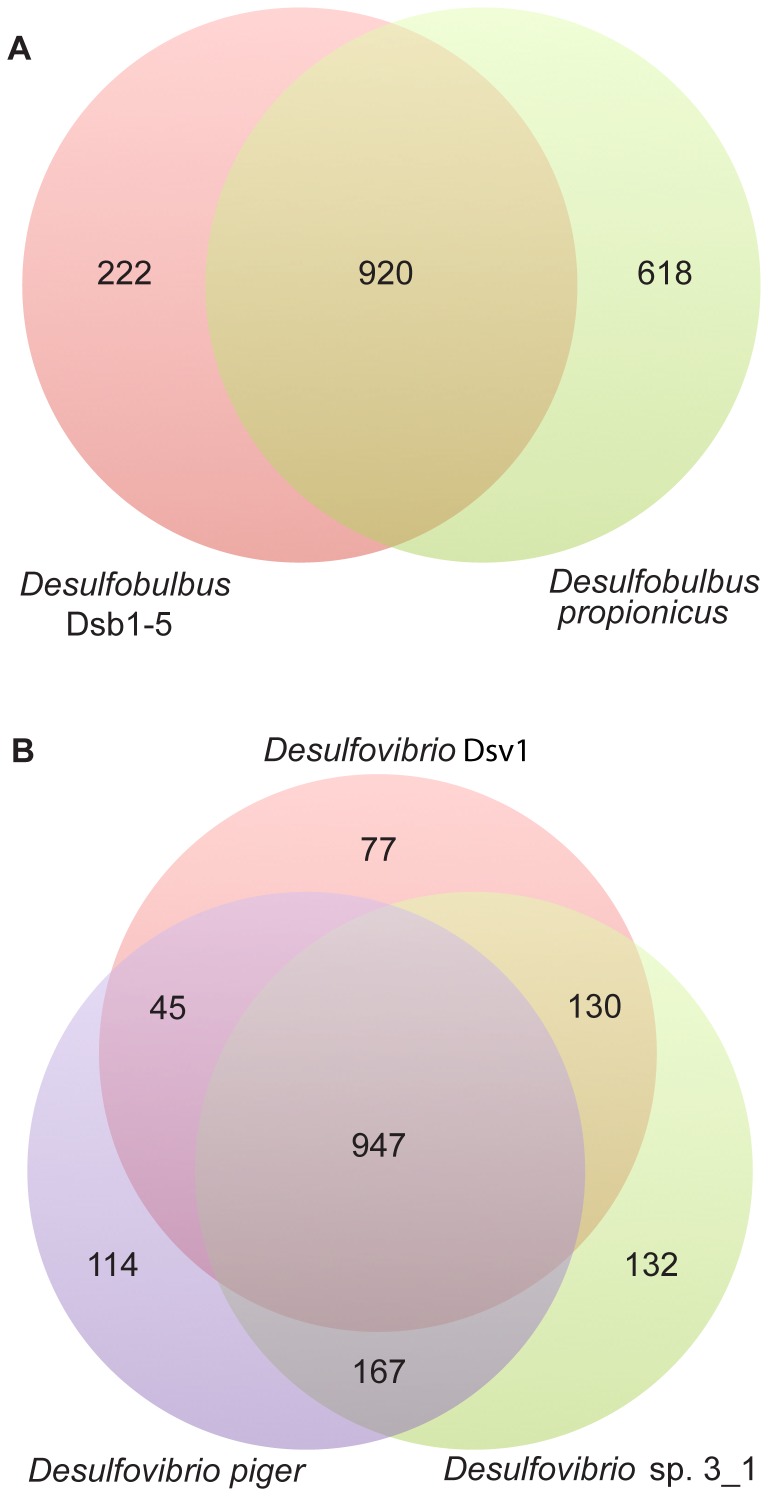
Comparison of COG categories (presence/absence) for human-associated and free living *Deltaproteobacteria*. (A). *Desulfobulbus* Dsb1-5 and *Desulfobulbus propionicus*. (B) *Desulfovibrio* Dsv1, *D*. sp. 3_1_syn3 and *D. piger*.

To further understand the unique properties of the oral *Deltaproteobacteria* characterized here, comparisons were done with other sequenced *Deltaproteobacteria* using the presence/absence of COGs. Overall percentages of genes in each COG category for Dsb1-5 and Dsv1 were similar to those seen in other sequenced *Deltaproteobacteria*. Comparisons of Dsb1-5 to *D. propionicus* closely matched initial comparisons made with BLASTP hits and revealed a large overlap of genes. Dsb1-5 shares 920 COGS with *D. propionicus*, whereas only 222 COGs (19%) were unique to the oral genomes (Figure 2A). A complete Dsb1-5 genome would likely show an even greater overlap with *D. propionicus*. Several of the unique COGs in Dsb1-5 were for related functions. Nine COGS included components of a type IV secretion system found in Dsb1-5, whereas only components of the type II secretion system were found in *D. propionicus*. Dsb1-5 also encodes an array of unique COGS for transporters, including those that transport dipeptides/oligopeptides, bacteriocins/lantibiotics, monosaccharides, betaine and cobalt. Dsb1-5 contained COGs for both Na^+^/alanine and Na^+^/H^+^-dicarboxylate symporters, while *D. propionicus* contains a unique multi-subunit, Na^+^/H^+^ antiporter. Many of the Dsb1-5 specific COGs contain proteins discussed above that may be used for host evasion and possible capsular production. Other differences include unique COGs in each genome for phage-related proteins and outer membrane proteins.

COG categories were also used to compare the oral Dsv1 genome to other available human-associated species, *D.* sp. 3_1_syn3 and *D. piger* (Figure 2B). Again, the COGs were similar to BLASTP results and largely overlapped, with 947 COGs found in all three species. An additional 130 COGs overlapped in both Dsv1 and *D.* sp. 3_1_syn3. COGs that were unique to Dsv1 included six NADH dehydrogenase subunits, a toxin/antitoxin growth regulation system, an ABC-type sulfate/molybdate transporter and an organic solvent tolerance protein. The large number of overlapping COGs found in all species included many metabolic genes, genes for cell division, DNA replication and repair and a large number of ABC-type transporters and Na^+^ symporters and antiporters.

A broader comparison of genomes within the *Desulfovibrio* genus found COGs that are specific to either host-associated or environmental organisms (Table S4 in File S1). Unfortunately, genes for four of the differential, human-associated COGs were predicted to encode uncharacterized proteins, leaving the function of these proteins unclear. Both pfam and top BLASTP hits for the uncharacterized COG proteins shed light on their potential function. The genes that fall into COG3735 also fall into pfam PF01963, the TraB family of proteins. In *Enterococcus faecalis*, TraB is an important regulator for the peptide pheromone cAD1 that is encoded on a hemolysin/bacteriocin plasmid and used to control both clumping and plasmid transfer between cells containing plasmids and those that do not [55]. COG2326-associated proteins are likely similar to polyphosphate kinase 2, an enzyme converts AMP to ADP. Another uncharacterized COG contained a protein that is found, in every host-associated genome analyzed, between a tripartite ATP-independent periplasmic (TRAP) transporter solute receptor protein and a TRAP fused permease component and may be involved in this secondary transport system. The final human-specific COG was a putative amino acid racemase.

In addition to these differential genes, eight additional genes fell into COG categories that were present in both human-associated and sheep rumen-associated *Desulfovibrio*, but absent in environmental *Desulfovibrio*. Interesting genes in this group include hits to COG2011 and COG1464. Further investigation of pfam and KO modules revealed these two proteins are likely used as a D-methionine transport system, and BLASTP results show that the closest hits outside of the host-associated *Desulfovibrio* are to clostridia. An additional COG (1897) that includes homoserine O-succinyltransferase, the first step in the biosynthesis of methionine, was found in all host-associated Deltaproteobacteria, except the incomplete, Dsb1-5 genome. Both COG0214 and COG0311 contain proteins that are part of the pyridoxine (vitamin B_6_) biosynthesis pathway.

Twenty-five COGs were detected only in the environmental *Desulfovibrio* species, eight of which are uncharacterized proteins. Two COGs encoded proteins similar to FtsE and FtsX, proteins involved in cell divison and important under low osmotic conditions [56]. Additional differential COGs include a distinct endocuclease III and IV and Fe-S oxidoreductase.

A comparison of all available *Deltaproteobacteria* genomes in IMG (*n* = 53) revealed two COG categories (COG4457, COG4458) that were found in all human-associated Deltaproteobacteria as well as *D. desulfuricans* ATCC 27774 but were not represented in any of the environmental Deltaproteobacteria genomes. These COGs included *srfB* and *srfC*, two genes of the SrfABC operon originally found in *Salmonella enterica* [*57*]. Although initially thought to be activated by SsrB, the transcriptional activator of a two-part regulatory system that controls gene activity of *Salmonella* pathogenicity island 2 (SPI2) [57], more recent work did not support this finding [58]. Additional studies have linked regulation of this operon to genes responsible for flagella production [59] and oxygen sensing [60]. Ultimately, the function of these genes is still unclear. BLASTP was used within IMG to find closely related predicted proteins in other genomes. When top hits were aligned and a phylogenetic tree was made, the most closely related proteins were found in a wide range of plant and animal-associated microbes, including both commensal and pathogenic bacteria (Figures S4, S5). Similar matches were found for a neighboring gene that encodes a putative *Haemophilus* adhesion and penetration (Hap)-like protein that is important for biofilm formation and adherence and entry into epithelial cell [61].

**Figure 3:**
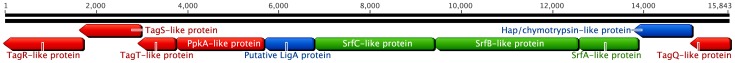
Gene neighborhood that includes two genes (*srfB* and *srfC*) unique to host-associated deltaproteobacterial genomes and with low similarity to *Deltaproteobacteria* from other environments. Genes are color-coded based on an association with the *srf* operon (green), type VI secretion (T6SS) post-translational control (red) or unknown association (blue). Genes encoding TagR, TagT and TagS were not found in Dsv1 or *B. wadsworthia*.

In addition to putative SrfABC and Hap encoding genes, all host-associated *Deltaproteobacteria* genomes share at least 3 other genes (*tagQ, ppkA and ligA)* in the same gene neighborhood. The majority of host-associated *Deltaproteobacteria* contained 3 additional homologous genes (*tagR*, *tagS*, *tagT*), resulting in a neighborhood of 10 genes with low homology to deltaproteobacterial genomes from other environments (Figure 3). Five of these genes (TagQ, TagR, TagS, TagT and PpkA) have been linked to type VI secretion (T6SS) post-translational control proteins in the threonine phosphorylation pathway (TPP) [62]. These genes act as part of a transmembrane signaling pathway that promotes T6SS activity under optimal conditions. Despite this, only *Desulfovibrio* sp. 3_1 contains genes necessary for type VI secretion machinery.

PpkA is a threonine protein kinase responsible for the phosphorylation of T6SS component Fha1, an event that triggers the export of an effector protein [62] Thus, it is possible that PpkA could trigger the export of SrfC, which was also predicted to be an effector protein [57]. TagQ is an outer membrane lipoprotein and is suspected to be involved in signal detection, due to its similarity to a 17kDa *Rickettsia* surface antigen [63]. TagR is proposed to be a co-receptor for the environmental signal that activates T6SS (Hsu, Schwarz et al. 2009). TagT and TagS form an ABC transporter that falls into a family of transporters that interacts with membrane-integrated histidine kinases and seem to play a role in regulated responses to external environment (Casabona, Silverman et al. 2012). All of the host-associated deltaproteobacterial genomes have TPP genes that are most closely related to those found in *Pseudomonas* species. This system in *Pseudomonas aeruginosa* has been shown to be an important virulence component [62].

One additional gene in this gene neighborhood encodes for a protein of unknown function. However, it was annotated as a LigA protein in at least one genome, and top BLASTP hits included *Taylorella asinigenitalis*, *Xenorhabdus bovienii* SS-2004, and *Proteus penneri* ATCC 35198, all host-associated organisms. Although it is remains unclear how these genes function, they are likely important to survival in the host and interactions with both host and other bacterial cells.

### Conclusion

**Figure 4:**
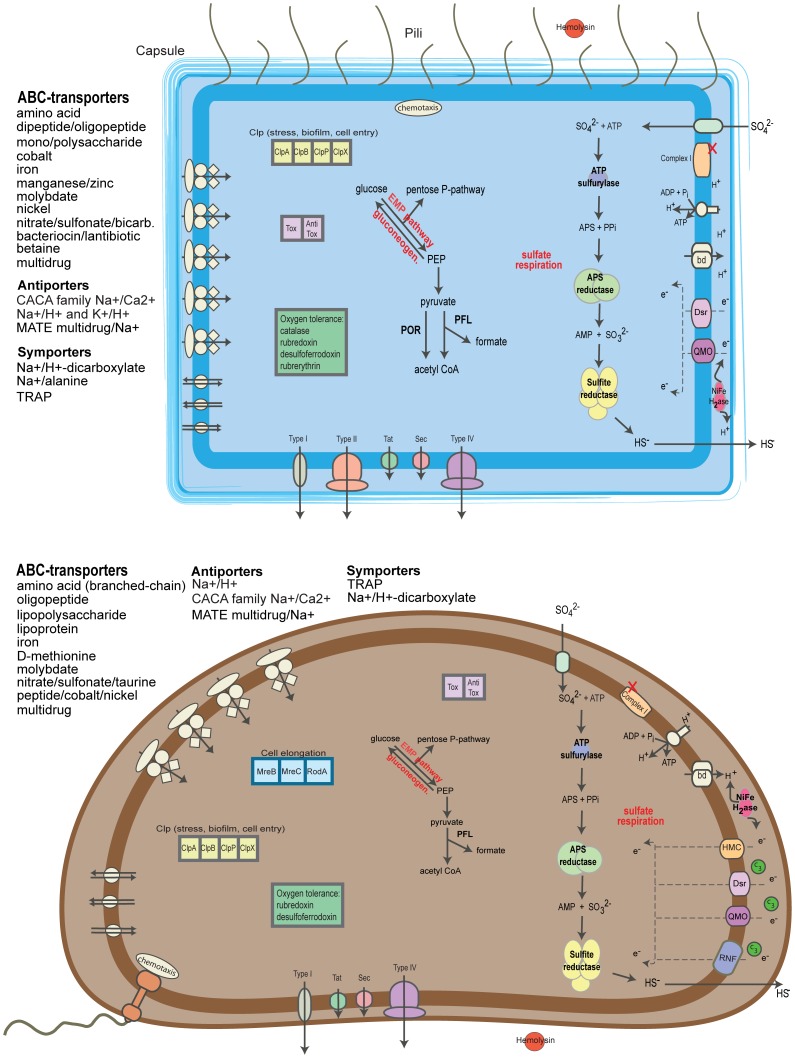
Brief overview of inferred metabolic and transport capabilities of *Desulfobulbus* Dsb1-5 (top panel) and *Desulfovibrio* Dsv1 (bottom panel). EMP, Embden-Meyerhof pathway. APS, adenosine 5′-phosphosufate. Bd, cytochrome bd. C_3_, cytochrome family III. Hmc, high-molecular-weight cytochrome c. Dsr, complex DsrMKJOP. QMO, QmoABC complex. RNF, Rnf-type complex I. Complex I, Nuo-type complex I (missing periplasmic components are denoted by a red X. H2ase, hydrogenase. PEP, phosphoenolpyruvatye. PFL, pyruvate formate lyase. POR, pyruvate ferredoxin oxidoreductase. TRAP, tripartite ATP-independent periplasmic. MATE, multidrug and toxic compound extrusion. Tox/AntiTox, killer gene system.

The genomes of Dsb1-5 and Dsv1 enabled the first insight into the potential functions of these *Deltaproteobacteria* within the oral environment (Figure 4). Even with an extreme environmental change, both groups still had considerable metabolic overlap with related environmental organisms. Although no other host-associated *Desulfobulbus* species have been sequenced, comparisons of Dsv1 with gut-associated *Desulfovibrio* revealed that there is good congruence in these genomes. However, it is clear that some oral and gut-associated species are more closely related than others (Dsv1 and *D.* sp_3_1_syn3 versus Dsv1 and *D. piger*). Further, analyzing distinct genes of Dsb1-5 and Dsv1 revealed a suite of genes that is essential for a host-adapted lifestyle. These unique genes are associated with adhesion, stress resistance, defense mechanisms and possible host-cell interactions and degradation. Thus, it is possible that associations of these organisms with disease are due to these virulent properties. This insight into the potential importance of uncultured, low-abundance members of microbial communities to disease will hopefully encourage similar genomic studies of other putative pathogens.

## Materials and Methods

### Ethics Statement

Human subjects enrollment and sample collection protocols were approved by the Ohio State University Institutional Review Board and by the Oak Ridge Site-Wide Institutional Review Board. Signed informed written consent was obtained from all human subjects that provided samples for this study.

### Sample Collection

We acquired three single-cells (Dsb1, Dsb2, Dsb3) and two combined pools (Dsb4, Dsb5) of a *Desulfobulbus* species, as well as one pool (Dsv1) of a *Desulfovibrio* species. Samples came from both periodontally healthy subjects and subjects with periodontitis. Patients were identified as having periodontitis based upon previously published criteria [16]. Dsb1 and Dsb2 were retrieved from a subgingival paper point sample taken from a healthy volunteer. Dsb3 was retrieved from a mixture of paper point samples taken from affected teeth from two subjects with periodontitis, and Dsb4 was retrieved from samples obtained by curette from a subject with periodontitis. Dsb5 and Dsv1 were retrieved from a mixture of paper point samples taken from affected teeth from four subjects with periodontitis. In all cases, samples were taken and immediately stored in 1× PBS at 4°C and transported on ice to the lab within 24 hours. When samples arrived at the lab, each was vortexed vigorously for one minute, and the PBS from each sample was combined as noted above. Samples were then fixed in 50% ethanol and stored at −20°C for one to three days before *in situ* hybridization.

### Fluorescence *in situ* Hybridization

For fluorescence *in situ* hybridization (FISH) we used the *Deltaproteobacteria*-specific probes DELTA495a [30] and SRB385 [29]. These are group-specific probes that have been shown to capture a large percentage of known *Desulfobulbus* and *Desulfovibrio* species [64]. Hybridization and wash buffers were prepared as previously described, and previous hybridization protocols were slightly adapted to work in-solution [65]. Briefly, 500 uL of ethanol-fixed samples were centrifuged (3000 g, 5∶00 min), and the resulting pellet was resuspended in 50 uL of a 1∶10 probe (50 ng/µL): hybridization buffer solution. Hybridization buffer contained 35% formamide [30]. This mixture was hybridized at 46°C for 3 hrs or overnight (∼20 hrs). Based on control hybridizations using *D. propionicus* and *D. piger*, the signal was enhanced by overnight incubations and that condition was applied to the oral samples. Following centrifugation, the cells were washed using standard FISH solution and resuspended in PBS for cell sorting. Both Alexa488-labeled DELTA495a probe alone and Alexa488-labeled DELTA495a mixed with Cy5-dual-labeled SRB385 [66] probe combinations were used. When samples were hybridized with the combined probes, cells had both red and green fluorescence.

### Cell Sorting

For cell sorting we used a Cytopeia Influx cell sorter (BD, Franklin Lakes, NJ, USA). The instrument was cleaned prior to use in a similar manner to those described earlier [67], [68]. Briefly, the fluidic lines were cleaned with 10% bleach for 45 min and rinsed with 0.2 µm filtered ddH_2_0 for 30 min, followed by rinsing with sheath fluid for an additional hour prior to sorting. 1× PBS was used as sheath fluid, and the reservoir tank and sheath fluid were UV-sterilized overnight. Between successive samples, fluidic lines were flushed with 10% bleach, followed by sheath fluid. Ethanol-fixed oral samples from periodontitis patients were first passed through a CellTrics 30 µm disposable filter (Partec, Görlitz, Germany). Prior to sorting, samples were labeled by FISH as described above, or stained with 5 µM of nucleic acid-binding dyes SYTO 9 (green) and SYTO 62 (red) (Life Technologies, Grand Island, NY USA) for 15 min. Cells labeled by FISH probes were sorted only once, as single cells. Samples labeled with nucleic acid dyes were sorted twice, using SYTO9 emission for the first sort and SYTO62 emission for the final sort, to further dilute any possible contaminating free DNA found in the original sample [68]. Cells sorted in the final round were deposited as single cells into 3 µL UV-sterilized TE buffer. Three *Desulfobulbus* cells (Dsb1, Dsb2, Dsb3) were obtained by random sorting using nucleic acid-binding dyes.

### Multiple Displacement Amplification

Single-cells retrieved by sorting were used for multiple displacement amplification (MDA). All plasticware, water and reagents used for MDA reactions (except the primers, dNTPs and enzyme) were UV treated as described [69]. Reactions were set up in a manner similar to [68]. Briefly, cells were subjected to lysis by addition of 3 µL of buffer that consisted of 0.13 M KOH, 3.3 mM EDTA pH 8.0 and 27.7 mM DTT, heated to 95°C for 30 sec, and immediately placed on ice for 10 min. 3 µL neutralization buffer (0.13 M HCl, 0.42 M Tris pH 7.0, 0.18 M Tris 8.0) was added followed by 11 µL of MDA master mix: 90.9 µM random hexamers with two protective, phosporothioate bonds on the 3′ end (Integrated DNA Technologies, Coralville, IA, USA), 1.09 mM dNTPs (Roche Indianapolis, IN, USA), 1.8× phi29 DNA polymerase buffer (New England BioLabs, Ipswich, MA, USA), 4 mM DTT (Roche) and ∼100 U phi29 DNA polymerase enzyme (purified in house) [70]. Amplification was performed for 10 hrs at 30°C followed by inactivation at 80°C for 20 min and storage at −20C.

### Target Identification

A small aliquot of each single cell amplified genomic product (SAG) was diluted (1∶150) in PCR-grade water (Ambion, Austin, TX, USA), and the remainder product was stored at −80°C. Dilutions were used for SSU rRNA gene PCR amplifications using universal bacterial primer 27fm (5′-AGA GTT TGA TYM TGG CTC AG-3′) [71] and *Deltaproteobacteria*-specific primer Delta495a (5'-AGT TAG CCG GTG CTT CCT-3′) (Loy, Lehner et al. 2002). Fifty microliter reactions contained the following: 1× polymerase buffer, 200 µM each dNTP (Roche, Indianapolis, IN USA), 2 mM MgCl2 (Ambion), 5 µg BSA (New England BioLabs), 300 µM each primer (Integrated DNA Technologies), 1 U of either *Taq* or *Pfu* polymerase and 1 µL diluted MDA product. PCR reactions ran under the following thermal conditions: 94°C for 2 min, followed by 30 cycles of 94°C for 30 sec, 55°C for 30 sec, 72°C for 1.5 min and a final extension at 72°C for 5 min. MDA products that gave amplicons were visualized by gel electrophoresis with 1% (w/v) agarose gels. All PCRs that produced clean bands were purified with PCR filtration plates (Millipore, Billerica, MA, USA) and sequenced directly on an ABI3730 DNA Analyzer (Applied Biosystems) using the primer 27 fm. Resulting chromatograms were manually edited and the sequences used for phylogenetic identifications with the RDP Classifier [72]. Samples for which the chromatograms were not homogeneous and that denoted potential heterogeneous template DNA (more than one sorted cell) were not further used. All confirmed deltaproteobacterial MDA products were used to generate secondary MDA products. Five secondary MDA reactions were set up for each SAG, and each reaction used 1 uL of a 1∶10 dilution of the original MDA product. All buffers and amplification mixes were constructed as described above for the primary MDA reactions. Secondary MDAs were only amplified for 6 hrs before heat inactivation.

### MDA Purification and Sequencing

Secondary deltaproteobacterial SAGs were purified by phenol:chloroform:isoamyl alcohol extraction and alcohol precipitation before being sent for sequencing. Illumina Hi-Seq 100-bp paired-end libraries were constructed and sequenced at the Hudson Alpha Institute for Biotechnology (Huntsville, AL, USA).

### Genome Assembly and Annotation

SAG libraries Dsb2 and Dsb3 were submitted to the Joint Genome Institute’s single-cell assembly and QC pipeline, similar to Illumina SAG libraries described previously [73]. Dsb1, Dsb4, Dsb5 and Dsv1 genomic data were normalized and assembled in-house using digital normalization [74] and Velvet assembly [75]. Briefly, for digital normalization, the following parameters were used: coverage threshold (C) = 30, k-mer size (k) = 30, n hashes (N) = 4 and minimum hash size (x) = 1e+09. In Velvet, each genome was computed from hash length 59 to 65 with a step of two. The number of nodes, n50, maximum number of bases in a scaffold and total number of bases for each hash length were compared to select the final assembled dataset. Assembled genomes were subjected to the JGI/ORNL automated annotation pipeline, which uses Prodigal for ORF calling [76], before submission into IMG [77] and RAST [78] for further analysis and for comparisons with genomes sequenced under the Human Microbiome Project [2]. The search for potentially contaminating DNA contigs (human and non-deltaproteobacterial) was conducted using BLASTP [32] as well as by GC content and tetramer analysis [79] of each scaffold. Contigs that were aberrant based on all these analyses were removed from further analysis but represented a small fraction of the dataset.

### Combined Non-redundant Assembly

To assess the phylogenetic relationships between the multiple Dsb SAGs, Dsb1-Dsb5 were compared using average nucleotide identity (ANI) and tetranucleotide frequency. Both ANI and tetranucleotide frequencies were calculated using the software JSpecies [80]. In order to discuss the potential function of oral *Desulfobulbus* as a whole, all datasets were combined (Dsb1-5). Repetitive regions between amplicons were removed iteratively with the aid of custom perl scripts. A region was considered redundant if it exhibited more than 95% identity over a region of at least 1000 bp. Remaining overlaps were assessed using BLASTCLUST [32] (S = 95, L = 0.7) as well as Mauve [81] and the *de novo* assembler within Geneious® Pro 5.6.5 and removed by hand. The final individual and combined assemblies were submitted to GenBank under BioProject accession numbers PRJNA188735-PRJNA188740. They are also readily available with full annotations under the public IMG portal at http://img.jgi.doe.gov/cgi-bin/w/main.cgi (taxon IDs: 2517572070, 3400000002, 3400000008, 3400000010–3400000012).

### Genome Size Estimation

Genome size and coverage was estimated using a conserved single copy gene (CSCG) set that has been determined from all 1516 finished bacterial genome sequences in the IMG database [77]. The set consists of 138 CSCG that were found to occur only once in at least 90% of all genomes by analysis of an abundance matrix based on hits to the protein family (Pfam) database [82]. Hidden Markov models of the identified Pfams were used to search assemblies by means of the HMMER3 software [83]. Resulting best hits above pre-calculated cutoffs were counted and the coverage was estimated as the ratio of found CSCG to total CSCGs in the set after normalization to 90%. Based thereon, the estimated complete genome size was calculated by division of the estimated genome coverage by the total assembly size.

## Supporting Information

Figure S1 **Optimization of sample hybridization times.** Panel A shows the scatterplot pattern of a *Desulfobulbus propionicus* culture after 3 (top) or 20 (bottom) hours of hybridization. Panel B shows actual sorting gates for an oral sample after 20 hour hybridization. All images of 528-38 emission (left) were samples hybridized with Alexa488-labeled DELTA495a. All images of 670-30 emission (right) were samples hybridized with Cy5-dual labeled SRB385.
(EPS)

Figure S2 **Number of Dsv1 genes with homologs in gut species** ***Desulfovibrio*** **sp. 3_1_syn3,** ***D. piger*** **or environmental species** ***D. vulgaris*** **Hildenborough.** The search for homologous genes was performed in IMG [85] with increasing minimum percent identity requirements. A total of 2890 Dsv1 genes were analyzed.
(PDF)

Figure S3 **Map of enzymes used in propionate metabolism and the methylmalonyl pathway.** Genes found in Dsb1-5 (based on E.C. number) are shown in blue. Genes in *Desulfobulbus propionicus* are shown in red.
(EPS)

Figure S4 **Maximum likelihood tree of putative** ***srfB*** **genes.** The tree was constructed using PHYML [86] in the program Geneious® Pro 5.6.5 with a JTT (+ gamma+invariant sites) substitution model. Predicted proteins from host-associated *Deltaproteobacteria* are denoted by a blue box. The scale bar indicates 0.3 substitutions per nucleotide position. Numbers given at the nodes represent bootstrap percentages calculated on 100 replicates.
(PDF)

Figure S5 **Maximum likelihood tree of putative** ***srfC*** **genes.** The tree was constructed using PHYML [86] in the program Geneious® Pro 5.6.5 with a JTT (+ gamma+invariant sites) substitution model. Predicted proteins from host-associated *Deltaproteobacteria* are denoted by a blue box. The scale bar indicates 0.8 substitutions per nucleotide position. Numbers given at the nodes represent bootstrap percentages calculated on 100 replicates.
(PDF)

File S1 (DOCX)
