# Supplementary figures and images for: Multiple Single-Cell Genomes Provide Insight into Functions of Uncultured Deltaproteobacteria in the Human Oral Cavity
Source: PLoS One. 2013 Mar 26;8(3):e59361. doi: 10.1371/journal.pone.0059361 (PMC3608642; doi:10.1371/journal.pone.0059361)

Number of Homologous Genes

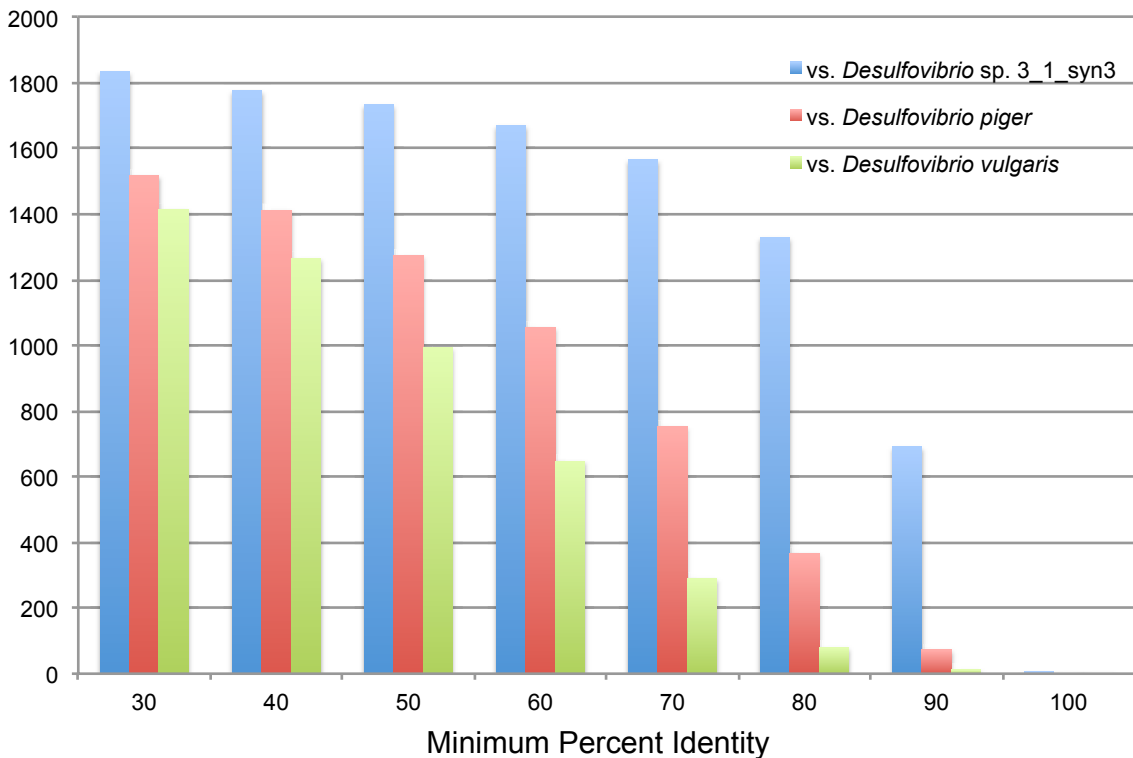

Supplement: Figure S2 — Number of Dsv1 genes with homologs in gut species Desulfovibrio sp. 3_1_syn3, D. piger or environmental species D. vulgaris Hildenborough. The search for homologous genes was performed in IMG [85] with increasing minimum percent identity requirements. A total of 2890 Dsv1 genes were analyzed. (PDF) [file pone.0059361.s002.pdf]

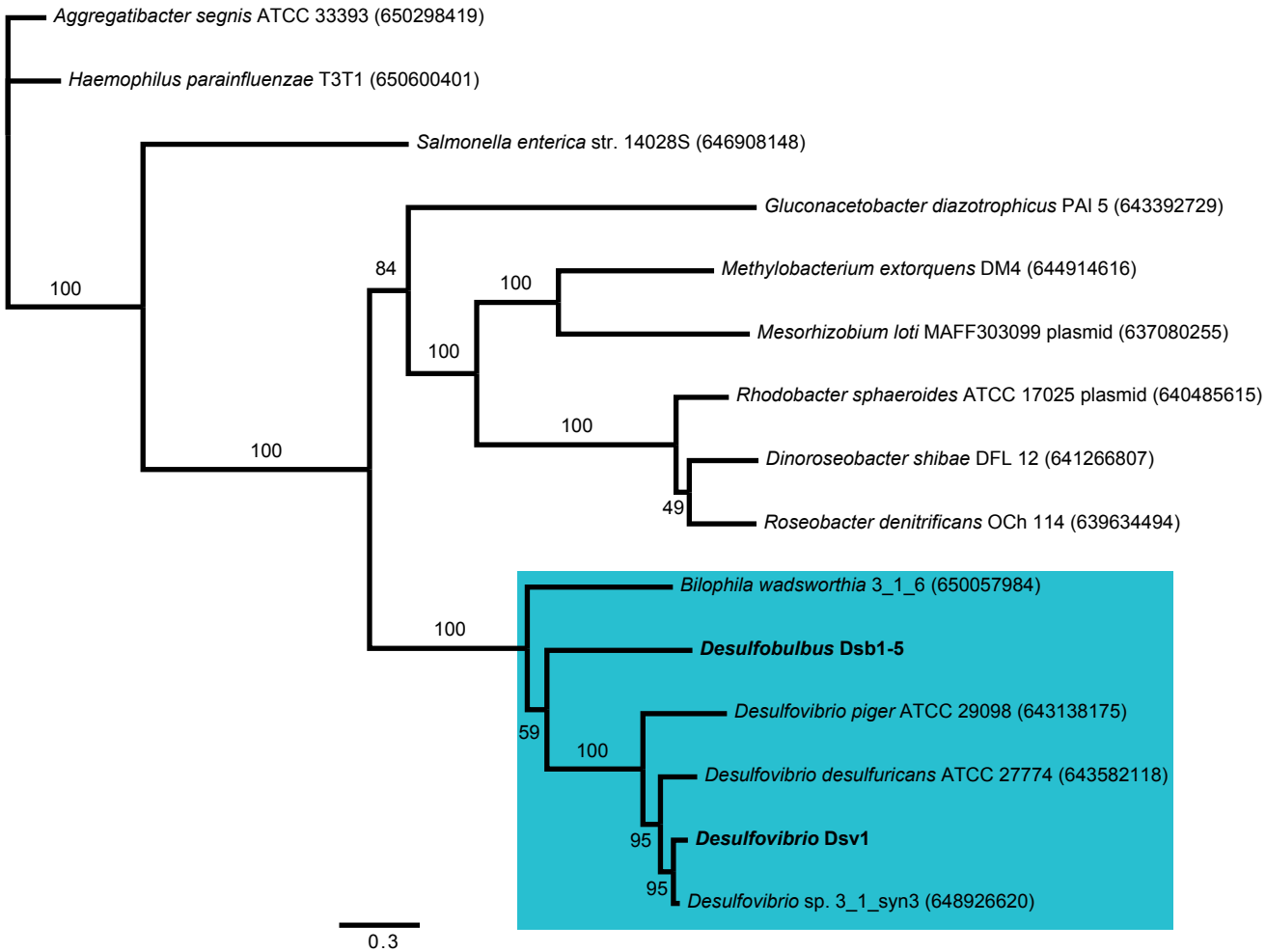

Supplement: Figure S4 — Maximum likelihood tree of putative srfB genes. The tree was constructed using PHYML [86] in the program Geneious® Pro 5.6.5 with a JTT (+ gamma+invariant sites) substitution model. Predicted proteins from host-associated Deltaproteobacteria are denoted by a blue box. The scale bar indicates 0.3 substitutions per nucleotide position. Numbers given at the nodes represent bootstrap percentages calculated on 100 replicates. (PDF) [file pone.0059361.s004.pdf]

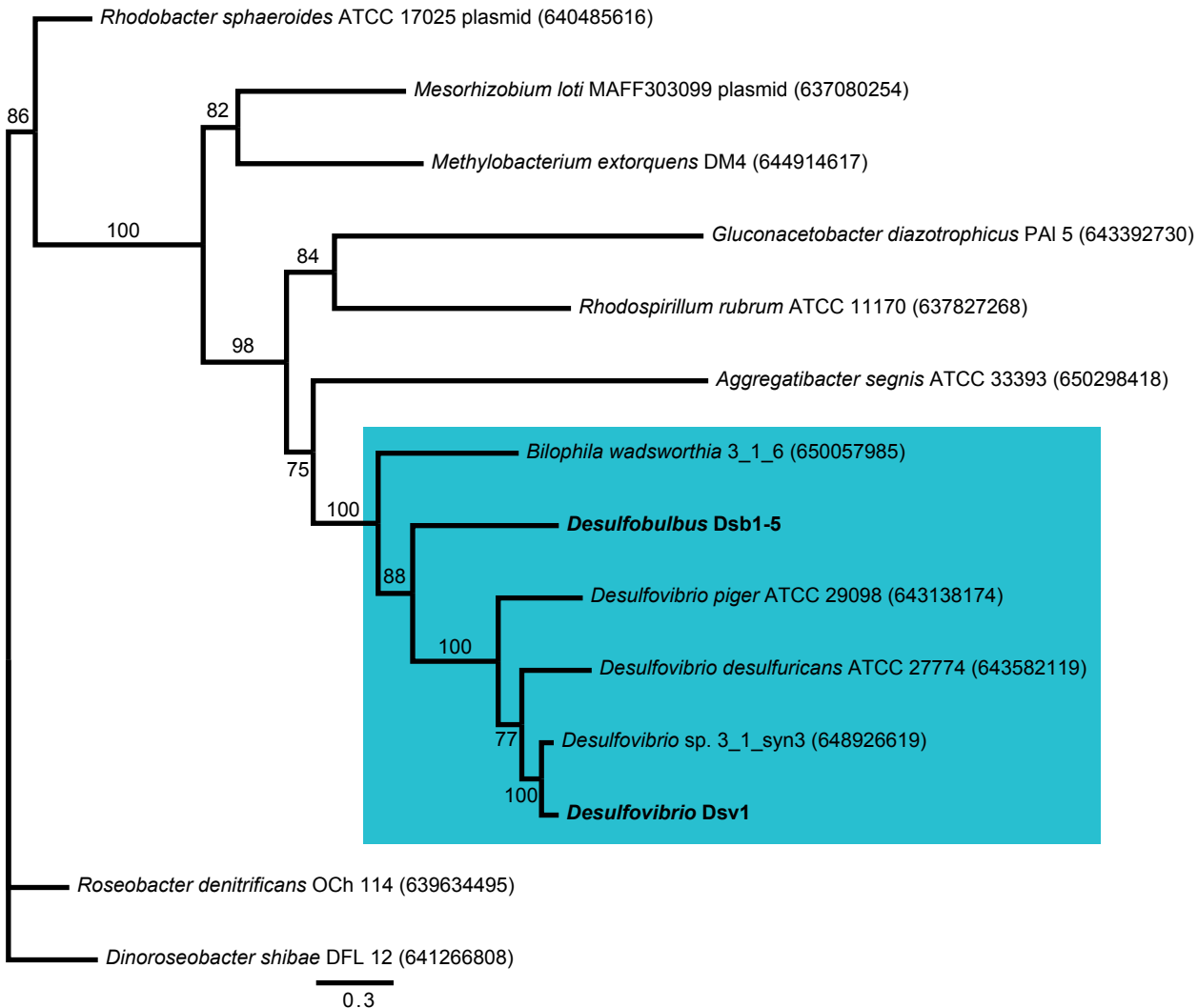

Supplement: Figure S5 — Maximum likelihood tree of putative srfC genes. The tree was constructed using PHYML [86] in the program Geneious® Pro 5.6.5 with a JTT (+ gamma+invariant sites) substitution model. Predicted proteins from host-associated Deltaproteobacteria are denoted by a blue box. The scale bar indicates 0.8 substitutions per nucleotide position. Numbers given at the nodes represent bootstrap percentages calculated on 100 replicates. (PDF) [file pone.0059361.s005.pdf]
