# Supplementary material for: Multiple Single-Cell Genomes Provide Insight into Functions of Uncultured Deltaproteobacteria in the Human Oral Cavity
Source: PLoS One. 2013 Mar 26;8(3):e59361. doi: 10.1371/journal.pone.0059361 (PMC3608642; doi:10.1371/journal.pone.0059361)
Supplement: File S1 — (DOCX) [file pone.0059361.s006.docx]

Table S1. Similarity matrix of *Desulfobulbus* genomes based upon average nucleotide identities.

|  | Dsb1 | Dsb2 | Dsb3 | Dsb4 | Dsb5 |
| --- | --- | --- | --- | --- | --- |
| Dsb1 | --- |  |  |  |  |
| Dsb2 | 99.11 | --- |  |  |  |
| Dsb3 | 98.00 | 97.79 | --- |  |  |
| Dsb4 | 97.02 | 96.62 | 96.89 | --- |  |
| Dsb5 | 95.86 | 96.03 | 97.60 | 97.72 | --- |

Table S2. Similarity matrix of *Desulfobulbus* genomes based upon tetranucleotide frequencies.

|  | Dsb1 | Dsb2 | Dsb3 | Dsb4 | Dsb5 |
| --- | --- | --- | --- | --- | --- |
| Dsb1 | --- |  |  |  |  |
| Dsb2 | 0.990 | --- |  |  |  |
| Dsb3 | 0.989 | 0.992 | --- |  |  |
| Dsb4 | 0.983 | 0.989 | 0.987 | --- |  |
| Dsb5 | 0.978 | 0.984 | 0.982 | 0.993 | --- |

Table S3. Dsb1-5 contigs that contain large numbers of horizontally acquired polysaccharide metabolism genes. Information for the top BLASTP hit for each predicted gene is shown.

| **Dsb1-5 Contig _Gene Number** | **Information for top BLASTP hit** | | | |
| --- | --- | --- | --- | --- |
|  | **E-value** | **Accession** | **Description** | **Organism** |
| Dsb1-5_Contig22_1 | 1.24621E-09 | ZP_10104881 | glycosyl transferase family 2 | *Thiothrix nivea* DSM 5205 |
| Dsb1-5_Contig22_2 | 2.94302E-66 | ZP_08821249 | glycosyl transferase family 2 | *Thiorhodococcus drewsii* AZ1 |
| Dsb1-5_Contig22_3 | 0.00747914 | EGH05187 | 4-oxalocrotonate tautomerase | *Pseudomonas syringae pv. aesculi* str. 0893_23 |
| Dsb1-5_Contig22_4 | 7.16306E-44 | ZP_07333891 | conserved hypothetical protein | *Desulfovibrio fructosovorans* JJ |
| Dsb1-5_Contig22_5 | 3.55497E-08 | ZP_06298373 | hypothetical protein pah_c004o242 | *Parachlamydia acanthamoebae* str. Hall's coccus |
| Dsb1-5_Contig22_6 | 6.82515E-20 | YP_001230579 | Ig family protein | *Geobacter uraniireducens* Rf4 |
| Dsb1-5_Contig22_7 | 1.04521E-53 | YP_003809026 | acetyltransferase | *Desulfarculus baarsii* DSM 2075 |
| Dsb1-5_Contig22_8 | 9.87963E-24 | YP_003206524 | membrane protein | *Candidatus Methylomirabilis oxyfera* |
| Dsb1-5_Contig22_9 | 1.31344E-71 | YP_003809023 | hypothetical protein Deba_3076 | *Desulfarculus baarsii* DSM 2075 |
| Dsb1-5_Contig22_10 | 5.189E-140 | YP_003806580 | family 2 glycosyl transferase | *Desulfarculus baarsii* DSM 2075 |
| Dsb1-5_Contig22_11 | 1.70997E-69 | YP_645837 | glycosyl transferase family protein | *Rubrobacter xylanophilus* DSM 9941 |
|  |  |  |  |  |
| Dsb1-5_Contig5_1 | 4.105E-97 | YP_002354782 | acetyl transferase protein | *Thauera* sp. MZ1T |
| Dsb1-5_Contig5_2 | 1.40426E-95 | ZP_10662483 | glycosyl transferase (involved in LPS synthesis) | *Pseudomonas* sp. GM48 |
| Dsb1-5_Contig5_3 | 2.8352E-158 | YP_728196 | glycosyl transferase | *Ralstonia eutropha* H16 |
| Dsb1-5_Contig5_4 | 1.8521E-158 | ZP_08931531 | UDP-N-acetylglucosamine 2-epimerase | *Thioalkalivibrio thiocyanoxidans* ARh 4 |
| Dsb1-5_Contig5_5 | 1.2898E-113 | YP_728198 | hypothetical protein H16_B0027 | *Ralstonia eutropha* H16 |
| Dsb1-5_Contig5_6 | 1.7469E-139 | ZP_09507281 | glycosyltransferase | *Marinobacterium stanieri* S30 |
| Dsb1-5_Contig5_7 | 2.2309E-168 | YP_003506150 | asparagine synthase | *Meiothermus ruber* DSM 1279 |
| Dsb1-5_Contig5_8 | 4.24594E-44 | ZP_04414198 | glycosyltransferase | *Vibrio cholerae* bv. *albensis* VL426 |
| Dsb1-5_Contig5_9 | 2.15494E-08 | ZP_08271641 | O-antigen polymerase precursor | gamma proteobacterium IMCC3088 |
| Dsb1-5_Contig5_10 | 0 | YP_983341 | LPS biosynthesis protein WbpG | *Polaromonas naphthalenivorans* CJ2 |
| Dsb1-5_Contig5_11 | 1.53953E-82 | YP_983344 | polysaccharide biosynthesis protein | *Polaromonas naphthalenivorans* CJ2 |
| Dsb1-5_Contig5_12 | 0 | YP_001339692 | DegT/DnrJ/EryC1/StrS aminotransferase | *Marinomonas* sp. MWYL1 |
| Dsb1-5_Contig5_13 | 2.5574E-107 | ZP_09911538 | hexapaptide repeat-containing transferase | SAR324 cluster bacterium JCVI-SC AAA005 |
| Dsb1-5_Contig5_14 | 0 | ZP_08931518 | oxidoreductase domain protein | *Thioalkalivibrio thiocyanoxidans* ARh 4 |
| Dsb1-5_Contig5_15 | 0 | YP_847464 | UDP-glucose/GDP-mannose dehydrogenase | *Syntrophobacter fumaroxidans* MPOB |
| Dsb1-5_Contig5_16 | 0 | YP_004194024 | polysaccharide biosynthesis protein CapD | *Desulfobulbus propionicus* DSM 2032 |
| Dsb1-5_Contig5_17 | 6.95693E-14 | CBL39994 | hypothetical protein CK3_01200 | butyrate-producing bacterium SS3/4 |
| Dsb1-5_Contig5_18 | 0.000524226 | ZP_08576528 | Gp54 protein | *Lactobacillus farciminis* KCTC 3681 |

Table S4. COGs unique to either human-associated or environmental *Desulfovibrio* genomes.

| **COGS unique to host-associated *Desulfovibrio*** | |
| --- | --- |
| COG0122 | 3-methyladenine DNA glycosylase/8-oxoguanine DNA glycosylase |
| COG1464 | ABC-type metal ion transport system, periplasmic component/surface antigen |
| COG2011 | ABC-type metal ion transport system, permease component |
| COG2759 | Formyltetrahydrofolate synthetase |
| COG3616 | Predicted amino acid aldolase or racemase |
| COG0311 | Predicted glutamine amidotransferase involved in pyridoxine biosynthesis |
| COG1058 | Predicted nucleotide-utilizing enzyme related to molybdopterin-biosynthesis enzyme MoeA |
| COG5496 | Predicted thioesterase |
| COG0214 | Pyridoxine biosynthesis enzyme |
| COG2326 | Uncharacterized conserved protein |
| COG4729 | Uncharacterized conserved protein |
| COG2315 | Uncharacterized protein conserved in bacteria |
| COG3735 | Uncharacterized protein conserved in bacteria |
|  |  |
| **COGS unique to environmental *Desulfovibrio*** | |
| COG1042 | Acyl-CoA synthetase (NDP forming) |
| COG1254 | Acylphosphatases |
| COG2177 | Cell division protein |
| COG0648 | Endonuclease IV |
| COG0731 | Fe-S oxidoreductases |
| COG0174 | Glutamine synthetase |
| COG1443 | Isopentenyldiphosphate isomerase |
| COG0346 | Lactoylglutathione lyase and related lyases |
| COG1762 | Phosphotransferase system mannitol/fructose-specific IIA domain (Ntr-type) |
| COG1075 | Predicted acetyltransferases and hydrolases with the alpha/beta hydrolase fold |
| COG2884 | Predicted ATPase involved in cell division |
| COG0702 | Predicted nucleoside-diphosphate-sugar epimerases |
| COG3635 | Predicted phosphoglycerate mutase, AP superfamily |
| COG5001 | Predicted signal transduction protein with membrane domain, an EAL and a GGDEF domain |
| COG1995 | Pyridoxal phosphate biosynthesis protein |
| COG1663 | Tetraacyldisaccharide-1-P 4'-kinase |
| COG0176 | Transaldolase |
| COG1690 | Uncharacterized conserved protein |
| COG1912 | Uncharacterized conserved protein |
| COG0011 | Uncharacterized conserved protein |
| COG0398 | Uncharacterized conserved protein |
| COG1900 | Uncharacterized conserved protein |
| COG2122 | Uncharacterized conserved protein |
| COG2509 | Uncharacterized FAD-dependent dehydrogenases |
| COG2231 | Uncharacterized protein related to Endonuclease III |
